# Youth overweight/obesity and its relationship with cardiovascular disease and parental risk factors

**DOI:** 10.20945/2359-3997000000156

**Published:** 2019-07-11

**Authors:** Martina Francesquet, Priscila Tatiana da Silva, Letícia de Borba Schneiders, João Francisco de Castro da Silveira, Silvana Silveira Soares, Debora Tornquist, Cézane Priscila Reuter

**Affiliations:** 1 Universidade de Santa Cruz do Sul Universidade de Santa Cruz do Sul Santa Cruz do Sul RS Brasil Universidade de Santa Cruz do Sul (UNISC), Santa Cruz do Sul, RS, Brasil; 2 Universidade de Santa Cruz do Sul Associação Pró-Ensino em Santa Cruz do Sul Universidade de Santa Cruz do Sul Hospital Santa Cruz Santa Cruz do Sul RS Brasil Programa de Residência Multiprofissional em Saúde, Associação Pró-Ensino em Santa Cruz do Sul/Universidade de Santa Cruz do Sul (APESC/UNISC), Hospital Santa Cruz, Santa Cruz do Sul, RS, Brasil; 3 Universidade de Santa Cruz do Sul Programa de Pós-graduação em Promoção da Saúde Universidade de Santa Cruz do Sul Santa Cruz do Sul RS Brasil Programa de Pós-graduação em Promoção da Saúde (Mestrado e Doutorado), Universidade de Santa Cruz do Sul (UNISC), Santa Cruz do Sul, RS, Brasil; 4 Universidade Federal de Pelotas Universidade Federal de Pelotas Pelotas RS Brasil Universidade Federal de Pelotas (UFPEL), Pelotas, RS, Brasil; 5 Universidade de Santa Cruz do Sul Programa de Pós-graduação em Promoção da Saúde Departamento de Educação Física e Saúde Universidade de Santa Cruz do Sul Santa Cruz do Sul RS Brasil Programa de Pós-graduação em Promoção da Saúde (Mestrado e Doutorado), Departamento de Educação Física e Saúde, Universidade de Santa Cruz do Sul (UNISC), Santa Cruz do Sul, RS, Brasil

**Keywords:** Adolescent, children, school, obesity, cardiovascular diseases

## Abstract

**Objective:**

The prevalence of overweight and obesity is gradually increasing in both developed and developing countries. Obesity, for instance, can present multifactorial causes that interact with each other. Among the important factors, parental obesity plays a prominent role in the onset of obesity during childhood and teenage years through genetics and ambient aspects. This study aims to verify the possible existence of an association between overweight/obesity of schoolchildren and cardiovascular risk (CVR) factors for their parents.

**Subjetcs and methods:**

For this purpose, a cross-sectional study was conducted with a sample of 1,243 children and adolescents, aged between 7 and 17. Out of the total number of participants, 563 (45.3%) were boys who were selected across 19 schools in the urban and rural areas of Santa Cruz do Sul, Rio Grande do Sul (Brazil). The overweight/obesity status of the schoolchildren was evaluated through their body mass index (BMI). Additionally, a self-reference questionnaire was employed to measure their parents’ CVR.

**Results:**

The results of this study revealed that students with overweight/obesity have a higher probability of having a father with hypertension (OR = 1.49; p = 0.038) and obesity (OR = 2.36; p = 0.002) and a mother with obesity (OR = 1.72; p = 0.016).

**Conclusion:**

To conclude, this study confirms a relationship between overweight/obesity of schoolchildren with CVR for their parents.

## INTRODUCTION

The prevalence of overweight and obesity is increasing in both developed and developing countries. The nutrition transition is characterized by a shift to diets rich in fat, sugar and processed foods but low in complex carbohydrates and fiber (1). However, in addition to dietary change, a variety of other interrelated variables contribute to obesity including metabolic, environmental, psychosocial and cultural factors. Individuals with a genetic predisposition for overweight are particularly susceptible to these factors (2).

Owing to its influence on both genetic and environmental variables, parental obesity is among the most important determinants of obesity in children and adolescents (3). More specifically, parental behavior influences children’s lifestyles, and this has been associated not only with obesity prevalence but with other cardiovascular risk factors. The sum of these risk factors has a larger effect on the onset and severity of cardiovascular disease than any one factor considered alone (4,5).

For these reasons, parents must be attentive to their own behavior as well as their children’s lifestyles. For example, long periods of sedentary activity and a failure to control one’s food intake increase the chances of developing obesity and cardiovascular disease earlier in life (6). The Bogalusa Heart Study points to the influence that environmental factors such as diet, smoking and physical activity have on hypertension and obesity (7). That study also suggests that personal habits learned in early life affect the onset of cardiovascular disease.

With respect to genetic factors, children and adolescents with two obese parents have an 80% chance of becoming obese, while those with only one obese parent have a 40% chance (8). Moreover, individuals can inherit a genetic predisposition for cardiovascular risk factors such as hypertension, dyslipidemia, diabetes and cardiovascular disturbances (9). Therefore, the current study aims to test the association between excess weight in students and the presence of cardiovascular risk (CVR) factors in their parents.

## SUBJECTS AND METHODS

This study consisted of a cross-sectional survey with children and adolescents aged 7 to 17 years. The total sample included 1,243 participants, of which 563 were boys from 19 schools in urban and rural areas of Santa Cruz do Sul, Brazil. This study is a part of a larger survey approved by the ethics and research committee at the University of Santa Cruz do Sul (UNISC). That survey interviewed 1,963 students between 2011 and 2012. Students whose parents did not complete that survey were excluded from this study. A signed consent form was obtained from the parents or guardians of all student participants. In addition, participants over 12 signed assent forms.

Overweight and obesity were determined using body mass index (BMI), which is calculated with the formula: kg/m^2^ (2). Using age- and gender-specific growth curves from the Center for Disease Control and Prevention/National Center for Health Statistics (CDC/NCHS) (10), participants were classified into four categories: 1) low weight (< p5), 2) normal (≥ p5 and < p85), 3) overweight (≥ p85 and < p95) and 4) obesity (≥ p95). These categories were then collapsed into two levels for statistical analysis: 1) low weight/normal, and 2) overweight/obesity. Parental CVR factors were determined using a self-report questionnaire. The questionnaire included questions regarding hypertension, heart attack, stroke, circulatory disease, increased total cholesterol (TC) and obesity.

Statistical analyses were performed in SPSS v. 23.0 (IBM, Armonk, NY, EUA). In order to understand the cardiovascular profile of parents, descriptive statistics (frequency and percentage) were calculated. Logistic regression was used to test the association between overweight/obesity in students and CVR in parents. Results were considered significant when p < 0.05.

## RESULTS AND DISCUSSION

Overweight/obesity was present in 29.1% of students. As shown in Table 1, hypertension was the most prevalent CVR in fathers (13.8%). Smaller percentages of fathers exhibited increased TC (9.3%), obesity (5.4%) and circulatory disease (5.1%). Similarly, the most common CVR in mothers was hypertension (19.7%), but circulatory disease (8.4%), obesity (8.4%) and increased TC (7.3%) were also present. Moreover, schoolchildren with overweight/obesity were more likely to have a father with hypertension (OR = 1.49; p = 0.038), a father with obesity (OR = 2.36; p = 0.002) or a mother with obesity (OR = 1.72; p = 0.016).

**Table 1:** Prevalence of cardiovascular disease and associated risk factors of student’s parents

Risk factor	n (%)
Father with hypertension	
No	1,071 (86.2)
Yes	172 (13.8)
Mother with hypertension	
No	998 (80.3)
Yes	245 (19.7)
Father with heart attack history	
No	1,220 (98.1)
Yes	23 (1.9)
Mother with heart attack history	
No	1,230 (99.0)
Yes	13 (1.0)
Father with stroke history	
No	1,230 (99.0)
Yes	13 (1.0)
Mother with stroke history	
No	1,236 (99.4)
Yes	7 (0.6)
Father with circulatory disease	
No	1,180 (94.9)
Yes	63 (5.1)
Mother with circulatory disease	
No	1,035 (83.3)
Yes	208 (16.7)
Father with increased TC	
No	1,127 (90.7)
Yes	116 (9.3)
Mother with increased TC	
No	1,152 (92.7)
Yes	91 (7.3)
Obese father	
No	1,176 (94.6)
Yes	67 (5.4)
Obese mother	
No	1,139 (91.6)
Yes	104 (8.4)

TC: total cholesterol.

The prevalence of overweight and obesity in students was high (29.1%). These results are in line with an earlier study among children and adolescents from different regions of Brazil that found the prevalence of overweight and obesity to be approximately 30% (11). The percentage of American adolescents aged 12 to 15 years who carry excess weight is comparable (total, 30.3%; overweight, 20.6%; obesity, 9.7%) (12). In Mexico, however, the prevalence of overweight and obesity is higher in both boys (children, 33.7%; adolescents, 33.5%) and girls (children, 32.8%; adolescents, 39.2%) (13). In contrast, a multicenter cross-sectional study in France reported that the prevalence of overweight and obesity in children was only 14.7% and 6.1%, respectively (14).

This study found a significant association between overweight/obesity in students and having a father with hypertension (Table 2). This result is similar to a study performed with adolescents aged 11 to 17 years in Curitiba, Paraná, Brazil. More specifically, the authors found that 18.2% (CI 95% 15.2-21.6) of students had altered blood pressure. In addition, those for whom both parents had hypertension (OR 2.22; CI 95% 1.28-3.85) and a high waist circumference (OR 2.1; CI 95% 1.34-3.28) were more likely to exhibit altered blood pressure (15). An Italian study similarly found that 17.4% of children and adolescents had hypertension. Moreover, compared to normal weight students, the risk for hypertension (OR 4.22; 95% CI 2.56-6.93) and pre-hypertension (OR 4.20; CI 95% 2.82-6.26) was higher in obese students. That study also reported an association between hypertension in mothers and pre-hypertension in children (OR 1.96; CI 95% 1.15-3.36) (16). Overall, these studies support our results because they indicate that hypertension and obesity in children are associated with hypertension and obesity in parents (15,17). According to the World Health Organization (WHO), excess body mass is responsible for 20% to 30% of hypertension cases (18). Thus, our results are in line with the hypothesis that obesity and heredity are risk factors for hypertension.

**Table 2:** Relationship between overweight/obesity of schoolchildren with cardiovascular diseases and associated factors from their parents

	Overweight/ obesity	p
	
	OR (CI 95%)	
Father with hypertension		
No	1	0.038
Yes	1.49 (1.02-2.17)	
Mother with hypertension		
No	1	0.838
Yes	1.04 (0.75-1.44)	
Father with heart attack history		
No	1	0.478
Yes	0.69 (0.25-1.90)	
Mother with heart attack history		
No	1	0.479
Yes	1.54 (0.46-5.16)	
Father with stroke history		
No	1	0.858
Yes	1.18 (0.30-3.80)	
Mother with stroke history		
No	1	0.700
Yes	1.38 (0.26-7.16)	
Father with circulatory disease		
No	1	0.224
Yes	0.69 (0.37-1,25)	
Mother with circulatory disease		
No	1	0.385
Yes	0.85 (0.60-1.21)	
Father with increased TC		
No	1	0.181
Yes	0.72 (0.45-1.60)	
Mother with increased TC		
No	1	0.699
Yes	1.10 (0.67-1.80)	
Obese father		
No	1	0.002
Yes	2.36 (1.37-4.08)	
Obese mother		
No	1	0.016
Yes	1.72 (1.10-2.69)	

OR: odds ratio; CI: confidence interval; TC: total cholesterol.

Interestingly, in contrast to hypertension, this study did not find an association between high TC or circulatory disease in fathers and overweight/obesity or cardiovascular disease in students. However, this association was documented in other studies. For example, Elias and cols. found that high blood pressure and an altered lipid profile are more common in children with hypertensive parents (19). More research is necessary to understand the reason for the discrepancy between this study and previous work.

A study performed by Stunkard and cols. suggested that genetic factors are more important determinants of children’s BMI than environmental variables (20). A cross-sectional study performed in Germany with children aged 5 to 7 years reported a relationship between the BMIs of parents and the BMIs of children. That study further suggested that parental BMI explains 7.6% of the variation in children’s BMI. Indeed, children with at least one obese parent were more likely to have excess body weight than children with one overweight parent. Yet, Stunkard and cols. also demonstrated that a child’s BMI was more strongly related to the BMI of his mother than the BMI of his father (r = 0.254 vs. 0.159, p < 0.01) (21). That finding fits with the results from this study, which found a high prevalence of obesity among fathers (5.4%) as well as a high prevalence of overweight/obesity in schoolchildren (29.1%). An Australian study looking at genetic factors for obesity reported that 80% of children with two obese parents were also obese. If only one parent was obese, then the percentage dropped to 50%; it was reduced to only 10% when neither parent was obese (22). Therefore, family weight profiles should be considered when identifying children at risk for obesity and when developing preventive measures (21,22).

A recent cross-sectional study with schoolchildren aged 6 to 10 years reported that the prevalence of overweight/obesity in parents was higher among those with overweight/obese children than among those with normal weight children (p < 0.01) (23). In a study with Brazilian adolescents aged 15 to 18, Terres and cols. also found an association between overweight/obesity in adolescents and obesity in parents (24).

The association between BMI in parents and BMI in children has been reinforced by several studies. Data from a study in Greece with adolescents aged 9 to 13 years showed that maternal or paternal obesity significantly increased the chances of childhood obesity (OR 2.25; CI 95% 1.45-3.48 and OR 2.14; CI 95% 1.28-3.60). Among other risk factors, the study found that maternal smoking (OR 1.37; CI 95% 1.05-1.98) and low parental education were important predictors of childhood overweight and obesity. In addition, schoolchildren were less likely to be obese if their parents had more than 12 years of formal education 0.46 (CI 95% 0.26-0.83) to 0.52 (CI 95% 0.36-0.74). This hints at the multifactorial nature of obesity as well as the need to increase scholarly understanding of the condition (25).

An Italian study with children aged 8 to 9 years found that only 1.4% of obese children had normal weight mothers, while 30.3% had obese mothers. Moreover, 4.0% of obese children had normal fathers, while 23.9% had obese fathers (26). A study from China with schoolchildren aged 7 to 18 years also found an association between student overweight and parental obesity (27). A recent study in Italian schools investigated the maternal risk factors, whether biological or environmental, that might contribute to the early onset of obesity in children aged 9 to 14 years. The prevalence of overweight and obesity in that study was high (27.2 and 14.1%, respectively), with boys more likely to be overweight/obese than girls (p < 0.05). According to the authors, breastfeeding was a protective factor (OR 0.64; p < 0.0005), but birth weight ≥ 4 kg (OR 1.83; p < 0.05), having an overweight/obese mother (OR 2.33; p < 0.0001), having an obese father (OR 1.68; p < 0.0001) and having parents with low/medium levels of education (OR 1.72; p < 0.005) were all risk factors for childhood obesity (28).

The results presented here did not suggest a relationship between student BMI and a parental history of heart attack, stroke or circulatory disease. However, a study performed with children and adolescents with a family history of coronary artery disease found that 10.1% and 15.6% of subjects were overweight or obese, respectively (29). Another study conducted in children and adolescents with a family history of premature coronary artery disease (CAD) reported that 12.8% of children and adolescents exhibited a single risk factor for atherosclerosis. In addition, 14.6% had two risk factors, 12.8% had three risk factors and 0.9% had four risk factors associated with a family history of CAD (30).

A cross-sectional study performed in the United States with children aged 5 to 12 years reported a correlation between parents’ perception of their children’s risk factors for cardiovascular disease and those children’s actual risks. In total, 15.4% percent of parents said that hypertension was a risk factor, while 11.2% said that heart disease was a risk factor. In addition, compared to the parents of normal weight children, more parents of obese children believed that their children had a high risk of obesity-related health problems (31).

These findings reinforce the need to include parents and children in screening programs for risk factors related to excess body weight (31). According to Lavie and cols., studies that evaluate the impact of weight loss on the control of cardiovascular disease are extremely important; these studies help with prevention and have a good cost-benefit ratio. It is clear that multipronged interventions can reduce the consequences of obesity, especially in the treatment of cardiovascular disease (32). The inflammatory profile in patients with obesity, atherosclerosis and cardiovascular disease is associated with variables such as visceral obesity, insulin resistance and dyslipidemia. Monitoring of these conditions may offer an alternative method for the control and treatment of obesity (33).

According to Ortega and cols., maintaining good cardiorespiratory fitness is a satisfactory method for reversing the cardiovascular damage caused by obesity (34). In the same vein, one study that evaluated the effects of an intervention for fatty liver disease reported promising results. Thus, that study similarly reinforces the importance of interventions aimed at treating and preventing diseases in youth (35) Recent studies also indicate that incentives for regular physical activity act as a protective factor against cardiovascular disease risk, metabolic syndrome and altered blood pressure in schoolchildren (36).

This study has several limitations. Because this was a cross-sectional study, it is not possible to establish causal associations. In addition, the data on risk factors in parents and schoolchildren were obtained via self-report, and this creates the possibility for response bias. On the other hand, a strength of this study was its large sample size, which helps ensure the representativeness of the sample. Moreover, all statistical analyses included relevant control variables, reducing the possibility that the reported results overestimate the true population parameters.

In conclusion, the results of this study indicate that there is a relationship between overweight/obesity in schoolchildren and CVR factors in parents. Schoolchildren who are overweight or obese are more likely to have a hypertensive father and obese parents.
